# Intraindividual phenotyping of depression in high-risk youth: An application of a multilevel hidden Markov model

**DOI:** 10.1017/S0954579423000500

**Published:** 2023-05-23

**Authors:** Qimin Liu, David Cole, Tiffany Tran, Meghan Quinn, Elisabeth McCauley, Guy Diamond, Judy Garber

**Affiliations:** 1 Department of Psychology and Human Development, Vanderbilt University, USA; 2 Department of Psychological Sciences, College of William & Mary, USA; 3 Psychiatry and Behavioral Medicine, University of Washington, USA; 4 Counseling and Family Therapy, Drexel University, USA

**Keywords:** depression, developmental psychopathology, intraindividual differences, longitudinal, Markov processes

## Abstract

**Background:**

Traditionally, depression phenotypes have been defined based on *interindividual* differences that distinguish between subgroups of individuals expressing distinct depressive symptoms often from cross-sectional data. Alternatively, depression phenotypes can be defined based on *intraindividual* differences, differentiating between transitory states of distinct symptoms profiles that a person transitions into or out of over time. Such within-person phenotypic states are less examined, despite their potential significance for understanding and treating depression.

**Methods:**

The current study used intensive longitudinal data of youths (*N* = 120) at risk for depression. Clinical interviews (at baseline, 4, 10, 16, and 22 months) yielded 90 weekly assessments. We applied a multilevel hidden Markov model to identify intraindividual phenotypes of weekly depressive symptoms for at-risk youth.

**Results:**

Three intraindividual phenotypes emerged: a low-depression state, an elevated-depression state, and a cognitive-physical-symptom state. Youth had a high probability of remaining in the same state over time. Furthermore, probabilities of transitioning from one state to another did not differ by age or ethnoracial minority status; girls were more likely than boys to transition from a low-depression state to either the elevated-depression state or the cognitive-physical symptom state. Finally, these intraindividual phenotypes and their dynamics were associated with comorbid externalizing symptoms.

**Conclusion:**

Identifying these states as well as the transitions between them characterizes how symptoms of depression change over time and provide potential directions for intervention efforts

## Introduction

Depression is a heterogeneous condition, particularly with respect to its symptom presentation. Existing research on subtypes of depression has largely focused on disentangling interindividual heterogeneity of depression. Less examined is the intraindividual heterogeneity among depressive symptoms. If the symptoms of depression change from time to time within the same person, then intraindividual heterogeneity exists, and different latent *states* may underlie symptom presentation at different times. Identifying these latent states as well as the transitions from one state to another will allow us to map the course of depression over time. These latent states can be defined in terms of symptom profiles (i.e., probabilities of exhibiting different levels of various depressive symptoms). We regard these latent *states* as intraindividual phenotypes, which provide a novel and useful way of conceptualizing the longitudinal dynamics of depressive symptoms. In an at-risk adolescent sample with weekly data across 90 weeks, we identified intraindividual phenotypes via state-of-the-art statistical modeling. We also examined sociodemographic and psychiatric correlates of these states.

Expressions of depression are known to be heterogeneous across individuals (Fried & Nesse, 2015; Fried, 2015; Liu et al., 2023; Zimmerman et al., 2015). That is, different individuals can have different combinations of depressive symptoms at a given point in time. Examples of such interindividual phenotypes are atypical depression - characterized by mood reactivity, weight/appetite increase, hypersomnia, leaden paralysis, and interpersonal rejection sensitivity, and melancholic depression - characterized by loss of pleasure and reactivity to pleasurable stimuli, hopelessness, terminal insomnia (American Psychiatric Association, 2013). Applications of latent class analysis to identify depressive subtypes are common in the literature, although, results have been inconsistent and often focus on overall symptom severity as the distinguishing feature (Ulbricht et al., 2018). The clinical utility of such severity-based classification relative to a continuous index of depression severity, however, is unclear. Furthermore, theoretical phenotypes (e.g., melancholic subtype in DSM-5) based on symptom assessment on one occasion has yielded limited differences in medication responses (Arnow et al., 2015). The limitations of interindividual depression phenotypes echo Kraepelinian emphasis to shift from classification systems that are based on cross-sectional clinical snapshots to longitudinal disease processes (Heckers & Kendler, 2020). Therefore, the goal of the current study was to identify intraindividual phenotypes from multivariate symptom patterns based on multiple assessments over time.

In addition to theory based interindividual phenotypes as in DSM, researchers have used growth mixture models and latent class trajectory models to empirically derive interindividual depression phenotypes that models between-person differences in symptom development. For instance, phenotypes have emerged in studies examining trajectories of depressive symptoms in those recovering from traumatic injuries (deRoon-Cassini et al., 2010), adolescent victims of cyberbullying (Hill et al., 2017), and postpartum mothers (Barboza & Schiamberg, 2021). A challenge with growth mixture models is that they assume the sample of interest is from a population that can be characterized by a single set of parameters (e.g., trend and baseline) that remain invariant across time (Ram & Grimm, 2009). For example, if a person is classified into the phenotype with increasing depressive symptoms, growth mixture models cannot consider how a person’s phenotype may change into a stable or decreasing trend. The time-invariant phenotype identification ignores the potential situational fluctuation where depressive symptoms may differ not due to between-person differences but due to within-person changes.

Expressions of depression vary from time to time within the same individual (Zhang et al., 2018). For example, an individual at one time may endorse primarily cognitive-affective symptoms, but at other times report predominantly physical symptoms. An analogy to climate versus weather may be useful: Interindividual heterogeneity in depression can be likened to climate differences between different regions. Like climate, interindividual differences in depression are regarded as highly stable over time. Conversely, intraindividual heterogeneity in depression is more like weather, insofar as intraindividual differences in depression are state-like and can change over time. The focus of the current study is on *weather* (i.e., within-person changes in patterns of depressive symptoms) rather than climate (i.e., between-person differences in average depressive symptom combination).

Intraindividual heterogeneity in depressive symptoms also exists outside of depressive episodes. Individuals can experience periods in which symptoms of depression exist but do not meet diagnostic criteria for major depressive episode (MDE). Moreover, these subthreshold levels of symptoms may combine in particular ways. In other words, these subclinical states may be complex in ways that cannot be captured by a single measure at a single timepoint and may be better characterized by profiles of distinct depressive symptom combinations. Identifying potentially multiple depression-related states in an at-risk sample may be informative regarding the longitudinal structure of depressive symptoms.

Phenotypes that target intraindividual heterogeneity may have clinical utility. Whereas contemporary literature on depression has promoted dimensional conceptualizations of psychopathology (Forbes et al., 2016), sophisticated categorical classification systems may remain efficient for practitioners’ decision making (Haeffel et al., 2022). For example, intraindividual phenotypes can represent clinically relevant states that can signal when a particular intervention may be especially helpful for a particular individual. Modeling the temporal dynamics of intraindividual phenotypes may also allow interventions to be tailored based on one’s tendency to transition into or out of different intraindividual phenotypes. Additionally, establishing intraindividual phenotypes can allow intervention researchers to quantify the impact of their interventions on the temporal dynamics of psychopathology (e.g., the tendency to relapse back into a problematic state).

One way to identify intraindividual phenotypes is through hidden Markov modeling. Hidden Markov modeling estimates latent states that are inherently specific to each measurement occasion. People may transition between states over time, and some states may be more stable than others. Each state is associated with a distinct pattern of symptom probabilities. Across repeated manifestations of a particular state, the pattern of symptom probabilities remains the same; that is, the high or low probability of observing a given symptom is consistent. Latent transition analysis, a special case of hidden Markov models, has been applied to identify and characterize intraindividual phenotypes pertaining to depression. For example, intraindividual phenotypes have been differentiated with respect to sociodemographic characteristics, psychiatric comorbidities, and treatment responsivity (Catarino et al., 2020; Rodriguez-Quintana & Lewis, 2019; Simmonds-Buckley et al., 2021; Wainwright et al., 1997). Focusing on the intraindividual phenotypes can provide clinical researchers with individual-specific and just-in-time differential “diagnoses.” In turn, this could improve the reliability of diagnoses and the validity of treatment-related decisions.

Beyond the identification of intraindividual phenotypes, the dynamics of these intraindividual phenotypes can be determined by investigating *transitions* between latent states. Some intraindividual phenotypes are highly stable over time, and they are said to have high *inertia*. Other intraindividual phenotypes are much less stable, as people transition readily from one to another. These phenotypes have low inertia. Understanding the stability of intraindividual phenotypes can reveal dynamics that underlie the course of depressive symptoms. Moreover, determining the person-specific dynamics of intraindividual phenotypes can reveal the relation of depression dynamics to other clinical and developmental conditions, as well as nuances in sociodemographic differences. For example, focusing on adults, the longitudinal Zurich Study from 1988, 1999, and 2008 found sex differences in not only depression phenotypes but also in the transitions between phenotypes (Rodgers et al., 2014). Three phenotypes, identified by symptom severity and psychosocial characteristics, emerged from their study: severe typical, severe atypical, and moderate. Two years of child data revealed that the inertias of depression phenotypes were associated with language competence and academic performance (Herman et al., 2018). Thus, the transitions between and the inertias of intraindividual depression phenotypes are associated with various correlates and outcomes.

The dynamics of intraindividual phenotypes may also correlate with psychiatric comorbidities. For example, a depressed state with a high inertia may result in chronicity and recurrence of depression, which is associated with comorbid internalizing (e.g., Melton et al., 2016; Verduijn et al., 2017) and externalizing symptoms (e.g., Powell et al., 2021). That is, dynamics between intraindividual phenotypes may signal differential comorbid psychiatric symptoms, all contributing to overall psychiatric prognoses and outcomes. Moreover, considering comorbid diagnoses provides insight into conditions that may contribute to or be the result of the persistence of depressive states. Sometimes, the relation between comorbid disorders can be bidirectional (Jacobson & Newman, 2017; Jansson-Fröjmark & Lindblom, 2008). For example, symptoms of conduct disorder (CD) may result in repeated interpersonal loss or failure. This interpersonal loss may then precipitate the occurrence of a depressive episode. On the other hand, maladaptive cognitive styles resulting from a depressed state may be associated with aberrant social information processing that minimizes positive cues and misattributes neutral or negative cues as hostile, in turn contributing to CD symptoms (Dodge, 1993). Thus, understanding diagnostic correlates of the dynamics between intraindividual phenotypes can help us identify potential contributors to and consequences of depressive states. Clinically, this understanding can help identify targeted comorbidity assessments and personalized intervention.

The literature on intraindividual phenotyping in depression contains several limitations. First, most studies have involved yearly assessments of depression. The DSM-5 (American Psychiatric Association, 2013) diagnostic criteria suggests that symptoms must persist for a minimum of only 2 weeks for a diagnosis of major depression. Assessments limited to 2-week symptoms afford only a low-resolution look at the course of depression, potentially missing evidences of inertias and transitions between different states across time. In the current study, we obtained information on the weekly occurrence of depressive symptoms. Second, most longitudinal studies of depression have relatively few measurement occasions, a fact that greatly limits the cross-time generalizability and reliability of intraindividual phenotypes. In the current study, we used information on weekly symptoms across 90 weeks. Third, latent transition analysis makes the highly restricted assumption that transition probabilities and inertias are the same for all individuals. Thus, we used a multilevel approach to Markovian modeling that estimates transition probabilities and inertias for each individual.

**Figure 1.**
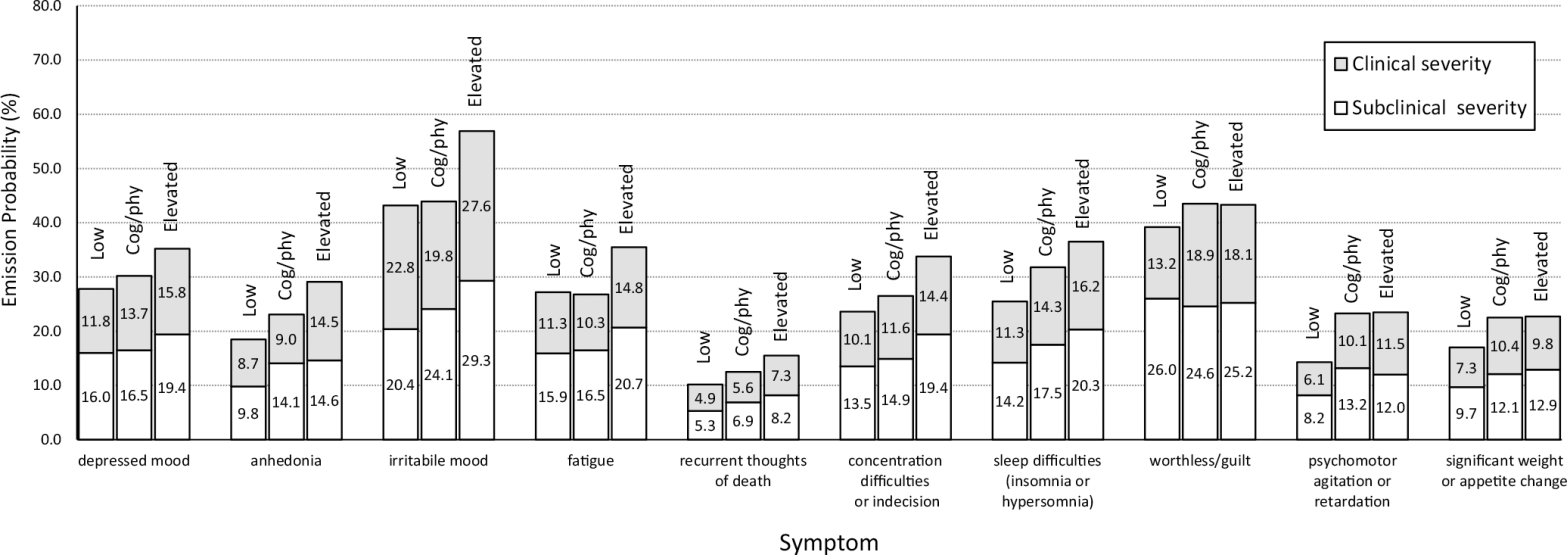
Emission probabilities (i.e., probabilities of endorsing symptoms at clinical or subclinical levels) for low-depression (low), elevated-depression (elevated), and cognitive-physical (Cog/Phy) states. **Note.** Numbers within each bars represent the probability of exhibiting the symptom at color-coded severity given state marked on top of each bars.

**Figure 2.**
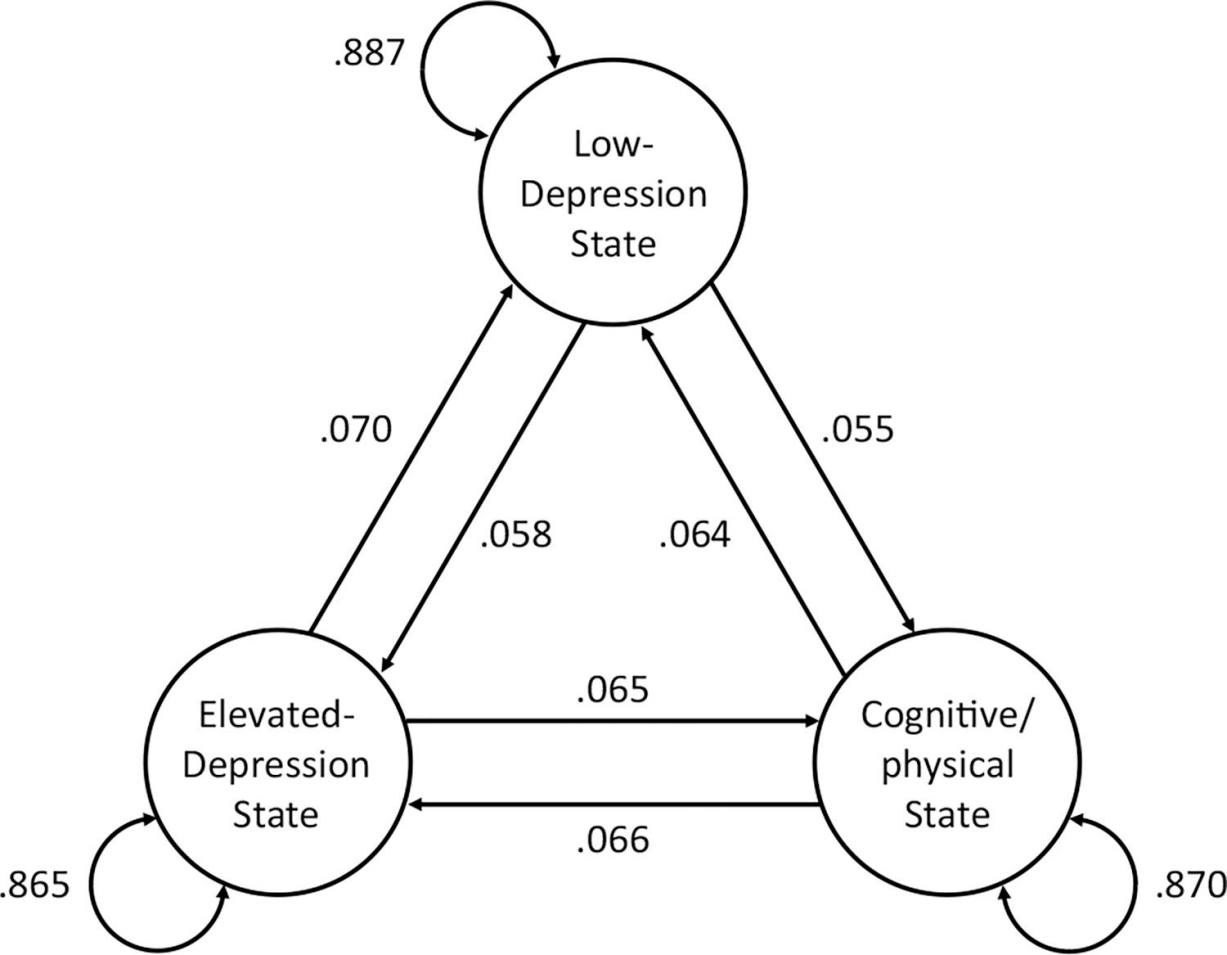
Transition probabilities and inertias of three-state multilevel hidden Markov model. **Note.** Each circle represents an identified intraindividual phenotype. Numbers on self-directed arrows represent inertias. Numbers on directed arrows between circles represent transition probabilities from the state an arrow points from to the state an arrow points towards.

Our study addresses these research gaps via an application of a multilevel hidden Markov model. This modeling approach helped us identify intraindividual phenotypes based on weekly depressive symptoms in at-risk youth. This approach had several advantages. First, it enabled us to examine the perseverance of latent states based on the pattern and severity of depression symptoms. Second, it offered sufficient measurement occasions for us to uncover intraindividual phenotypes that are generalizable and reliable. Third, multilevel hidden Markov models allowed us to account for individual differences in the probability of observing symptoms when in a particular state (i.e., *emission probabilities*: e.g., the probability of exhibiting sleep disturbance when in an elevated-depressed state; see Figure 1 for an illustration) and the probability of transitioning between states (i.e., *transition probabilities*: e.g., the probability of transitioning from a low-depression state into an elevated-depression state; see Figure 2 for an illustration). Finally, we also examined sociodemographic and psychiatric correlates of these individual differences in state dynamics to clarify the significance and implications of these intraindividual phenotypes. As our analyses were exploratory in nature, we did not make specific hypotheses. Our goal was to identify intraindividual phenotypes in youth and to examine how identified intraindividual phenotypes, as well as their dynamics, were related to sociodemographic and psychiatric characteristics.

## Method

### Participants and procedure

The current study used data of at-risk youth (*n* = 124) from a longitudinal study of offspring of treatment-seeking depressed parents (Garber et al., 2011). The primary purpose of the original study was to examine relations between changes in parents’ depression and their children’s psychopathology. Parents and children were interviewed about the child’s psychopathology at baseline and again at 4, 10, 16, and 22 months after the initial assessment. At each interview, participants retrospectively reported the children’s symptoms since the previous interview, yielding symptom data for 90 weeks. Over the 90 weeks, 42 youth experienced at least 1 week of subclinical levels of depression, 23 experienced at least 1 week of clinical levels of depression, and 70 did not experience subclinical nor clinical levels of depression. The current analyses used ratings combined from parent and child reports of Garber and colleagues’ (2011) study.

Four participants (3%) were excluded due to substantial missingness (>80%) on study variables; observations with missing values on studied variables were removed (3%). The final analytic sample consisted of 120 participants with a total of 10,346 observations. Youths ranged in age from 7 to 17 years old (mean = 12.26, SD = 2.35). The youth participants were 53.3% female, 68.3% Caucasian, 22.5% African American, 2.5% Hispanic or Latino, 0.8% Asian, and 8.3% multiracial.

### Measures

The Longitudinal Interval Follow-up Evaluation (LIFE; Keller, 1987) was administered to parents and youths about the child’s depressive symptoms since their last assessment. The LIFE is a reliable and valid, interview-based assessment of clinical symptoms and diagnoses. The interviewers linked symptoms to important events such as the start of school, birthdays, and holidays, etc. This approach is considered the gold standard for longitudinal assessments as it improves symptom recollection. Since its creation, the LIFE has been used in several studies (e.g., Nogami et al., 2022; Vittengl et al., 2022) and continues to be administered to investigate change in psychiatric symptoms. Data for the present analyses were obtained from the LIFE and included the DSM’s symptoms of depression for youth according to the DSM: anhedonia, depressed mood, irritable mood, sleep difficulties (insomnia or hypersomnia), fatigue, significant appetite or weight change, concentration difficulties or indecision, psychomotor symptoms (agitation or retardation), worthlessness or excessive guilt, and recurrent thoughts of death or suicide. Symptoms were rated on 3-point severity scales (1 = absent, 2 = subclinical, 3 = clinical) and were assessed following standard guidelines for measuring symptoms in youth (Kaufman et al., 1997). In addition, the interviews inquired about symptoms of generalized anxiety disorder (GAD), separation anxiety disorder (SAD), attention deficit hyperactivity disorder (ADHD), oppositional defiant disorder (ODD), and CD during the same intervals. Each disorder was coded from 1 to 6 based on the number of symptoms and level of impairment (1 = no symptoms, 2–3 = subclinical symptoms and mild interference, 4 = 4 subclinical symptoms and moderate impairment, 5 = 5 or more clinically significant symptoms and moderate impairment, 6 = 6 or more severe clinically significant symptoms and impairment, suicide attempt, or hospitalization). Interviews about youth symptoms were conducted separately for parent and child. Their reports were combined using the “or” rule: If either the parent or the child indicated that a symptom was present, then it was marked as present. However, if the interviewer determined that one informant was not likely to have information about a child’s symptoms (e.g., the child was away for several weeks), then greater weight was given to the other informant. For instance, if a parent reported that their child had sleep problems during a week the child was at summer camp and the child reported no sleep problems during that time, then sleep problems would be rated as not present for that week. Inter-rater reliability for all diagnoses yielded *κ*s ≥ .78 (depression = .82; anxiety disorders = .78; externalizing disorders = .85).

### Data analysis

We applied multilevel hidden Markov models (Maruotti et al., 2021) via R package “mHMMbayes” (Aarts & Moraga, 2022) to identify latent *states*, characterized by different constellations of depressive symptoms. Hidden Markov models assume latent states that are associated with distinct probabilities of experiencing depressive symptoms (i.e., emission probabilities). Furthermore, hidden Markov models allow opportunity to identify probabilities for remaining in a hidden state or to transition between hidden states (i.e., transition probabilities). Multilevel specification further allows modeling individual differences in both emission and transition probabilities. The model is described further in the supplementary material. We tested models with 2–10 states and selected the final model based on Akaike information criteria (AIC), where smaller AICs indicate better fit after accounting for model complexity (Akaike, 1974).

For the final model, we computed average emission probabilities (i.e., the conditional probabilities that each symptom was absent, subclinical, or clinical for youth in an underlying latent state). We also obtained a matrix of average transition probabilities (i.e., the probabilities of transitioning from one latent state to another). The diagonal elements of the transition probability matrix represent the inertia of the state (with higher inertia values signifying a greater tendency to remain in a particular state). We also computed the average number of weeks for the first-time passage from one latent state to a different latent state (first-passage time), as well as average number of weeks for a given latent state to recur (recurrence time). We estimated sex and age differences on these transition probabilities using multivariate linear regression.

In addition, we applied multilevel models with random intercepts and lag-1 autoregressive residuals to investigate the relation of latent states and inertias to comorbid symptom severities. More specifically, after controlling for sex and age, we examined the association of state inertia to the severity of ADHD, CD, GAD, ODD, and SAD. To control the family-wise Type-I error rates for these five models, we used a Bonferroni corrected nominal alpha level of .05/5 models for 5 comorbid psychiatric disorder = .01.

## Results

The three-state model provided the best fit to the data (AIC = 452.50).^1^ To characterize these states, we plotted the symptom profiles for each state. Specifically, Figure 1 shows the emission probabilities of being at the clinical or subclinical level of each symptom for each state, (i.e., p(clinical or subclinical | state)). The first was a low-depression state, characterized by relatively low emission probabilities for all depressive symptoms at the clinical or subclinical level. The second was an elevated-depression state, characterized by relatively high emission probabilities for all depressive symptoms. The third state was a cognitive-physical symptoms state, characterized by emission probabilities (generally as large as those for the elevated-depression state) elevated particularly for cognitive and physical symptoms. The cognitive-physical symptoms state was not simply a “medium-depression” state that exhibited medium level emission probabilities across all symptoms. Rather, youths in the cognitive-physical states were comparable to those in elevated-depression state for worthlessness/guilt, psychomotor disturbance, and weight/appetite disturbances. They were comparable to those in low-depression state for irritable mood and fatigue while displaying medium emission probabilities for other symptoms at subclinical/clinical levels.

In Figure 2, the curved double-headed arrows depict the average inertia probabilities for each state, and the straight single-headed arrows depict the average transition probabilities from one state to another. All three states exhibited high inertia between measurement occasions. Specifically, the probability of remaining in the same state week-to-week was 86.5% for elevated-depression, 88.7% for low-depression, and 87.0% cognitive-physical symptoms states. The transition probabilities from the low-depression state to other states were lower than those in the reverse directions. The transition probability from cognitive-physical to elevated-depression states was comparable to the reverse transition probability from the elevated-depression state to the cognitive-physical state. On average, transitions from the elevated-depression state to low-depression and cognitive-physical states took 14.7 and 16.3 weeks, respectively. Transitions from the low-depression state to elevated-depression and cognitive-physical states took 16.6 and 17.2 weeks, respectively. Transitions from the cognitive-physical states to elevated-depression and low-depression states took 15.8 and 15.1 weeks, respectively. On average, state recurrence took 3.2 weeks for the elevated-depression state, 2.7 for the low-depression state, and 3.2 for the cognitive-physical state.

Table 1 shows the relations of the transition probabilities to demographic characteristics. Girls were more likely than boys to transition from low-depression to elevated-depression states (standardized *β* = .44, *p* = .02) and cognitive-physical states (*β* = .45, *p* = .02). Girls were more likely than boys to transition out of the low-depression state (*β* = −.44, *p* = .02). Other transition probabilities showed no sex differences. Neither age nor ethnoracial minority status was associated with any transition probabilities.

Table 2 presents the relation of states and state inertias to other forms of psychopathology. The inertia to stay in an elevated-depression state was positively associated with comorbid ADHD symptom severity. Individuals in concurrent elevated-depression or cognitive-physical states (compared to a low-depression state) had less severe CD symptoms. Inertia for all states was positively associated with ODD symptom severity. In addition, compared to the low-depression state, the elevated-depression state was associated with severe ODD symptoms, and the cognitive-physical state was associated with mild ODD symptoms. States and state inertias were not associated with anxiety disorders after multiplicity corrections.

## Discussion

The current study yielded four major findings. First, we identified three intraindividual phenotypes of weekly depressive symptoms among at-risk youth: elevated-depression, low-depression, and cognitive-physical *states*. Second, youth had a strong tendency to remain in the same state over time (i.e., all identified intraindividual phenotypes showed high state inertias). On average, these states took 3 weeks to recur and 16 weeks to transition into other *states*. Third, sex was associated with transitions between intraindividual phenotypes, whereas age and ethnoracial minority status were not. Specifically, girls were more likely than boys to transition out of the low-depression states into elevated-depression states and cognitive-physical states. Lastly, intraindividual phenotypes and their inertias were associated with comorbid externalizing but not internalizing symptoms.

First, across 90 weeks, depressive symptoms among at-risk youths were characterized by three intraindividual phenotypes: (1) an elevated-depression state where the probability of endorsing each depressive symptom at either the subclinical or clinical level was high; (2) a low-depression state where the probability of endorsing all depressive symptoms at *nonclinical* levels was high; and (3) a cognitive-physical state where the probability of observing subclinical or clinical levels of worthlessness/guilt, appetite/weight disturbance, sleep difficulties, and psychomotor disturbance, were comparable to those of the elevated-depression state and substantially higher than those of the low-depression state. In the cognitive-physical state, the probability of subclinical or clinical levels of depressed mood, anhedonia, concentration and indecision, and thoughts about death was lower than in the elevated-depression state. The phenotypes that we identified from youths differ from Rodgers and colleagues’ (2014) work with European adults. Our phenotypes revealing symptom-level nuances also differ from those described by Herman and colleagues (2018), which focused on overall depression severity ratings. The classification of intraindividual phenotypes paves a new path in the evaluation and treatment of depression. To meet DSM criteria for a diagnosis of a depressive episode, depressive symptoms must occur together for a period of at least 2 weeks or more. Traditional depression diagnoses can implicitly assume that symptoms presented at the time of the interview are representative and stable without the utilization of empirical data on intraindividual symptom changes. Intraindividual phenotypes integrate the dynamic associations of depressive symptoms on a weekly basis, enabling clinicians to continuously evolve their differential diagnosis and subsequent treatment plan.

The cognitive-physical state echoes prior findings on the physiological underpinnings in negative cognitive appraisal (Gartland et al., 2014). Existing research on psycho-neuroendocrinology has documented strong relations between cognitive and physical functions (McEwen & Akil, 2020). According to an information processing framework, depression is associated with both selective attention to negative information and exaggerated threat processing (Ingram et al., 2007; Scher et al., 2005). These biased cognitive processes may activate the amygdala and the hypothalamic-pituitary-adrenal (HPA) axis, leading to increased allostatic load (Gaab et al., 2005; McEwen & Gianaros, 2011). Persistent activation of the HPA axis is implicated in physiological consequences including immune malfunction, disease susceptibility, and cardiovascular risks (Cohen, 2021; Reiche et al., 2004; Rosmond & Bjorntorp, 2000).

Second, the intraindividual phenotypes that we identified were relatively stable (i.e., had a high degree of inertia). That is, youths who were in a particular state tended to remain in that state. On average, roughly 28.8% of the sample was in elevated-depression state, 43.2% of the sample were in low-depression state, and 37.2% of the sample were in the cognitive-physical state. On the one hand, inertia of the low-depression state is consistent with the finding that only up to about 35% of offspring of depressed parents develop depression themselves during adolescence (Weissman et al., 2021); that is, some at-risk children remain in nondepressed states. On the other hand, the high inertia of the elevated-depression and cognitive-physical states highlights the importance of intervention, as these states are unlikely to change on their own. Our findings expand prior research on the overall stability of depressive symptoms over longer time intervals (Lovibond, 1998; Morken et al., 2021; Zuroff et al., 1999) and align with studies that have reported high stability among symptom states of depression in youth (Catarino et al., 2020; Simmonds-Buckley et al., 2021). This description of stability suggests potential variability in responses to therapy amongst various symptom states as a valuable next step to inform treatment decisions and optimize cost-effectiveness. Relatedly, the average number of weeks to transition from one state to another was 16. On average, it took 14.7 weeks to transition out of elevated-depression and 15.2 weeks to transition from cognitive-physical states to the low-depression state. Notably, the elevated-depression state has similarities to a MDE, where the median episode duration has found to be 16 weeks for children, 8 weeks for adolescents, and 12 weeks for adults (Rohde et al., 2013).

Estimates of state duration could be used in the evaluation of interventions, insofar as effective interventions should result in more rapid than typical transition out of depressed states. In the current sample, the average number of weeks for any of these states to recur was approximately 3 weeks; that is, youth tended to revisit their elevated-depression or cognitive-physical states in about 3 weeks. Similarly, low-depression youths were likely to return to their previous low-depression state in about the same amount of time. This suggests that it might be prudent to re-assess depression in youth after about 3 weeks post-recovery to evaluate relapse.

Third, transitions from low risk to cognitive-physical and elevated-depression states were more likely to occur in girls than boys. Research has clearly documented that girls are more likely to be diagnosed with a depressive disorder, to experience depressive symptoms, and to exhibit an increasing trend in depression severity (Essau et al., 2010; Lewis et al., 2020; Parker et al., 2014; Salk et al., 2017). Interesting to note is that the severity of depression has not been found to differentiate between girls and boys in a depressive episode (Bennett et al., 2005; Hankin et al., 1998; Parker et al., 2014). The current results are consistent with previous findings that the rates of depression increase faster in girls more than boys beginning in early adolescence (Hyde et al., 2008).

The sex difference in these transition probabilities revealed nuances on the higher prevalence of depression in girls than boys. Not only were girls more likely than boys to experience depression, but girls were more likely to *shift* into depression-related states and less likely to remain in low-depression states. This may be partially due to the fact that girls experience more stress exposure such as peer stress, body dissatisfaction associated with societal gender expectation, and family stress (Hankin et al., 2015; Hankin & Abramson, 1999; Nolen-Hoeksema, 2001). Prior research has also revealed that youth with more depressive episodes have earlier depression onset, and, for girls, earlier onset predicts a worse course of depression (Essau et al., 2010). Thus, our findings provide further support for the importance of targeting girls for prevention of depression (Strauman et al., 2011). Sex differences were not observed, however, in the transition probabilities from high risk and cognitive-physical states to other states. This echoes extant research finding lack of gender differences in recovery from depression (Wei et al., 2021).

Fourth, the intraindividual phenotypes identified here were associated with comorbid externalizing symptoms. Youth who were more likely to remain in an at-risk state tended to have more severe ADHD symptoms. Indeed, ADHD often preceded the onset of depression (Kessler et al., 2005). The challenges of ADHD likely remain unchanged, thereby prolonging high risk for youth depression (Riglin et al., 2021). Thus, not only did depressive symptoms and ADHD symptoms co-occur, but youth with ADHD were more likely to have chronically elevated levels of depressive symptoms. This highlights the need to monitor youth with ADHD for depressive symptoms and to intervene early.

Results showed that being in an elevated-depression state or cognitive-physical state was associated with fewer symptoms of CD. Children with CD symptoms tend to be characterized by limited prosocial emotions, lack of remorse or guilt, callousness, and affect deficiency (American Psychiatric Association, 2013). Such features are quite distinct from symptoms of depression, raising the possibility that the processes underlying certain depressive states may be incompatible with those that result in CD. Conversely, the cognitive-physical phenotype and individual differences in its inertia were associated with more symptoms of ODD, potentially suggesting that the processes underlying the cognitive-physical depressive phenotype may complement those that underlie ODD.

This study advanced the existing literature on depression phenotyping by examining intraindividual heterogeneity in depressive symptoms among at-risk youth. Prior studies have been limited to grouping patients into symptom-based immutable classes. Distinct from these efforts, the current study identifies states characterized by probabilities of endorsing depressive symptoms while demonstrating the temporal nature of these depressive states over time. In at-risk youth specifically, findings suggest evidence for the existence of differential intraindividual phenotypes, indicated by different symptom levels across states and probabilities of transition between states.

We caution that phenotypes are not prescriptive of what underlies the co-occurring symptoms but rather they are one way to descriptively characterize symptom co-occurrences. Choosing to describe depression as a sequence of state phenotypes can have pragmatic advantages. Practitioners are unlikely to be able to change intervention strategies in response to every slight variation in a continuous severity. Far more efficient would be for practitioners to make adjustments in treatment plans when their clients are seen as shifting from one depressive state to another. Knowing whether depression in nature changes dimensionally or categorically over time may be challenging. Whereas a dimensional view-point will be more useful for some purposes, a categorical approach can be more useful for others.^2^ Particularly, our identification of categorical states using a probabilistic approach based on empirical data can be particularly advantageous.

Our empirical phenotyping bases category assignment on more information, generating a richer probabilistic understanding that can influence treatment, compared to using predetermined thresholds on a continuous scale. Our empirically derived phenotyping makes probabilistic predictions of an individual’s likelihood to be in each state by incorporating symptom-level information. Consider this imaginary scenario: Alex’s symptom presentation at time A might suggest that they are 55% likely in the low-depression state, 25% in the cognitive-physical state, and 20% in the elevated-depression state. However, at time B, Alex’s symptom presentation might suggest that they are 55% likely in the low-depression state, 5% in the cognitive-physical state, and 40% in the elevated-depression state. Such a probabilistic approach first allows us to identify Alex as being in the low-depression state at both times, helping us allocate treatment resources to others with higher needs. Secondly, even though Alex would be in a low-depression state in both scenarios, probabilistic information can alert us to Alex’s relatively heightened risk to elevated-depression in the second scenario. This may indicate a need for a preventive intervention. Our approach contrasts with category identification using severity-only information: because Alex scores 2 out 9 on depression severity, Alex would be considered nondepressed in both time A and B. Moreover, the overall severity rating does not pertain to specific symptom combinations that allow treatment personalization or provide information on Alex’s risk levels towards elevated depression. Additionally, through intraindividual phenotyping, practitioners can provide model-assisted guesses on patient’s future probabilities of being in certain states using transition probability estimates. This would not be possible with conventional phenotyping based on interindividual criterion of depressive episodes.

Limitations of the current study suggest directions for future research. First, our assessments of youth symptoms were retrospective; as such, they may have been affected by recall bias. Future studies could use ecological momentary assessment strategies to measure symptoms, although participant burden might reduce the quality of the data over time. Second, the current study focused on at-risk offspring of parents with current depression. Future studies may seek to examine the intraindividual phenotypes of depression in depressed or healthy youth with or without a family history of depression. Examining other samples can help determine whether the identified phenotypes replicate and generalize to new and different samples, and if additional phenotypes emerge. Third, the current study did not have information on treatment (i.e., psychotherapy or pharmacotherapy). Future studies may seek to obtain this information to understand possible variation in state stability and transitions. Future studies can incorporate intraindividual phenotyping to understand the impact of interventions on state stability and transitions. Fourth, our method assumes invariance in week-by-week intraindividual dynamics between identified phenotypes, although we did not find evidence of between-person age differences. Future research should consider potential developmental changes especially with studies following youth across larger age span. Furthermore, intensive longitudinal research designs would enable future researchers to use models that account for developmental shifts such as regime-switching hidden Markov models.

## Conclusion

In conclusion, the current study identified intraindividual phenotypes underlying depressive symptoms of at-risk youth across 90 consecutive weeks. These phenotypes were characterized by elevated-depression, low-depression, or cognitive-physical symptoms; all phenotypes were relatively stable. Average time to reach other states or revisit the current state indicates the importance of evaluating depressive symptoms at various points over time. Girls were more likely to transition out of low-depression states and into one of the two higher-elevated states; therefore, targeted prevention efforts for at-risk girls especially may be in order. Finally, intraindividual phenotypes of depressive symptoms were associated with comorbid externalizing symptoms, suggesting that assessment and interventions for at-risk youth presenting with symptoms of depression also should evaluate for symptoms of other disorders in addition to depression.

## Supplementary Material

1

## Figures and Tables

**Table 1.** Sex, age, and ethnoracial differences in state transition probabilities

	*b*	95 % *CI*	*t*	*p*	*b*	95 % *CI*	*t*	*p*	*b*	95 % *CI*	*t*	*p*
Predictors	Elevated-depression → Elevated-depression				Elevated-depression → Low-depression				Elevated-depression → Cognitive-physical			
Female	0.02	[−0.02, 0.06]	1.08	0.28	−0.01	[−0.03, 0.01]	−1.10	0.27	−0.01	[−0.03, 0.01]	−1.04	0.30
Age	0.00	[−0.01, 0.01]	0.68	0.50	0.00	[−0.01, 0.00]	−0.78	0.44	0.00	[−0.00, 0.00]	−0.59	0.55
Ethnoracial minority	0.00	[−0.04, 0.04]	0.00	>.99	0.00	[−0.02, 0.02]	−0.08	0.94	0.00	[−0.02, 0.02]	0.13	0.90
Predictors	Low-depression → Elevated-depression				Low-depression → Low-depression				Low-depression → Cognitive-physical			
Female	**0.02**	**[0.00, 0.03]**	**2.36**	**0.02**	**−0.04**	**[−0.08, −0.01]**	**−2.37**	**0.02**	**0.02**	**[0.00, 0.04]**	**2.44**	**0.02**
Age	0.00	[−0.00, 0.00]	0.43	0.67	0.00	[−0.01, 0.01]	−0.40	0.69	0.00	[−0.00, 0.00]	0.43	0.67
Ethnoracial minority	0.00	[−0.02, 0.01]	−0.51	0.61	0.01	[−0.03, 0.05]	0.58	0.56	−0.01	[−0.03, 0.01]	−0.81	0.42
Predictors	Cognitive-physical → Elevated-depression				Cognitive-physical → Low-depression				Cognitive-physical → Cognitive-physical			
Female	0.01	[−0.01, 0.03]	1.24	0.22	0.02	[−0.01, 0.04]	1.54	0.13	−0.03	[−0.08, 0.01]	−1.45	0.15
Age	0.00	[−0.01, 0.00]	−1.57	0.12	0.00	[−0.01, 0.00]	−1.67	0.10	0.01	[−0.00, 0.02]	1.59	0.11
Ethnoracial minority	0.01	[−0.01, 0.03]	1.09	0.28	0.01	[−0.01, 0.04]	1.16	0.25	−0.03	[−0.07, 0.02]	−1.07	0.29

**Note.** *b* = unstandardized regression coefficients, *CI* = confidence interval; significant findings at .05 α level are bolded.

**Table 2.** Associations of depressive states and state inertias to comorbid symptom severity

Predictors	*b*	SE	t	p	95% CI LL	95% CI UL
			Attention deficits hyperactivity disorder			
Elevated-depression state	0.01	0.014	0.47	0.639	−0.021	0.035
Cognitive -physical state	−0.01	0.015	−0.44	0.657	−0.036	0.023
**Female**	**−0.86**	**0.247**	**−3.50**	**0.001**	**−1.353**	**−0.376**
Age	−0.07	0.051	−1.43	0.155	−0.173	0.028
**Elevated-depression state inertia**	**4.19**	**1.167**	**3.59**	**0.001**	**1.876**	**6.499**
Low-depression state inertia	1.54	1.383	1.11	0.267	−1.198	4.281
Cognitive -physical state inertia	2.33	1.078	2.16	0.033	0.196	4.468
			Conduct disorder			
**Elevated-depression state**	**−0.03**	**0.011**	**−2.88**	**0.004**	**−0.051**	**−0.010**
**Cognitive -physical state**	**−0.03**	**0.011**	**−3.15**	**0.002**	**−0.056**	**−0.013**
Female	0.12	0.143	0.86	0.394	−0.161	0.406
Age	0.06	0.029	1.92	0.057	−0.002	0.115
Elevated-depression state inertia	1.70	0.677	2.51	0.014	0.357	3.040
Low-depression state inertia	−0.10	0.803	−0.12	0.904	−1.687	1.493
Cognitive -physical state inertia	0.54	0.626	0.86	0.390	−0.699	1.780
			Oppositional defiant disorder			
**Elevated-depression state**	**0.09**	**0.020**	**4.39**	**0.000**	**0.048**	**0.125**
**Cognitive-physical state**	**−0.07**	**0.021**	**−3.56**	**0.000**	**−0.114**	**−0.033**
Female	−0.50	0.196	−2.54	0.013	−0.888	−0.110
Age	0.00	0.040	−0.03	0.978	−0.081	0.079
**Elevated-depression state inertia**	**5.14**	**0.929**	**5.53**	**0.000**	**3.302**	**6.983**
**Low-depression state inertia**	**3.63**	**1.101**	**3.29**	**0.001**	**1.445**	**5.807**
**Cognitive-physical state inertia**	**3.29**	**0.858**	**3.84**	**0.000**	**1.593**	**4.993**
			Generalized anxiety disorder			
Elevated-depression state	−0.03	0.015	−2.39	0.017	−0.063	−0.006
Cognitive state	−0.04	0.015	−2.50	0.013	−0.068	−0.008
Female	−0.09	0.133	−0.69	0.490	−0.357	0.172
Age	0.02	0.027	0.86	0.393	−0.031	0.078
Elevated-depression state inertia	1.31	0.630	2.08	0.040	0.062	2.560
Low-depression state inertia	1.33	0.747	1.78	0.078	−0.153	2.806
Cognitive-physical state inertia	1.26	0.582	2.17	0.032	0.110	2.416
			Separation anxiety disorder			
Elevated-depression state	−0.01	0.009	−1.66	0.097	−0.032	0.003
Cognitive-physical state	−0.02	0.009	−2.50	0.013	−0.041	−0.005
Female	0.03	0.075	0.45	0.654	−0.115	0.183
Age	−0.02	0.015	−1.57	0.120	−0.055	0.006
Elevated-depression state inertia	0.34	0.355	0.97	0.335	−0.360	1.047
Low-depression state inertia	0.01	0.421	0.04	0.972	−0.818	0.848
Cognitive-physical state inertia	0.36	0.328	1.11	0.270	−0.286	1.013

**Note**. *b* = unstandardized regression coefficients, *SE* = standard error; significant findings at .01 α level are bolded.

## Footnotes

1 AIC from all models with the number of states from 2 to 10 were 490.34, 452.50, 503.94, 557.64, 638.86, 704.82, 781.44, 869.96, 948.167.

2 Of note is that the complementary relation between categorical and dimensional views reflects their underlying statistical equivalence: Statistical models that seek to discover categorical phenotypes and those that seek to identify dimensional constructs from observed symptoms can yield the same observed relations between symptoms (Gibson, 1959; Halpin et al., 2011; Lubke & Neale, 2006; McDonald, 1967; Molenaar & von Eye, 1994; Steinley & McDonald, 2007).
