# Supplementary material for: Intraindividual phenotyping of depression in high-risk youth: An application of a multilevel hidden Markov model
Source: Dev Psychopathol. Author manuscript; Available in PMC 2024 Nov 23. (PMC10665546; doi:10.1017/S0954579423000500)

**Multilevel hidden markov model for modeling intraindividual depression phenotype**

We observed depression symptoms of an individual over many measurement occasions. Hidden Markov models assume that observed depressive symptoms at a given measurement occasion reflect a hidden state (i.e., intraindividual phenotype). By knowing the underlying hidden state of an individual at a measurement occasion, one is able to infer the probability of an individual exhibiting a depressive symptom at a certain level, i.e., emission probability. Furthermore, hidden Markov models assume that the current hidden state can be related to the past hidden state. By knowing the current state, one is able to infer the probability of an individual being in a hidden state in a future measurement occasion, i.e., transition probability.

The hidden Markov model thereby estimates two main sets of parameters of interest as applied to our data: (1) a 3-dimensional (10 symptoms (depressed mood, anhedonia, irritable mood, fatigue, recurrent thought of death, concentration difficulties or indecision, sleep difficulties, worthlessness/guilt, psychomotor disturbances, weight/appetite disturbances) by 3 symptom levels (nonclinical, subclinical, clinical) by *K* states) emission probability array (2) a transition probability matrix.

Multilevel hidden Markov model can be expressed as the likelihood to observe the symptoms *X* from the second measurement occasion and onwards is

$$f\left( x | \pi\right)=\prod_{t=2}^{T} \prod_{n}^{N} \prod_{i}^{S} \prod_{j}^{S} \pi_{ijn}^{{[s}_{tn}=j]\cdot[s_{(t-1)n}=i]}$$

Here T represents the number of measurement occasions, N represents the number of participants, S represent state (e.g., $s_{tn}$ is the realized state for individual *n* at measurement occasion *t*. The transition probability from state *i* to *j* for individual *n* is denoted as $\pi_{ijn}$. Given categorical observed variables *X,* the emission probability for individual *n* of observing symptom *p* (e.g., depressed mood) at level *o* (e.g., nonclinical, subclinical, clinical) for individual *n* at time *t* is dependent on the hidden state (for individual *n* at time *t*) $S_{tn}$ that and follows a categorical distribution: $\Pr(X_{tnp}=o\mid S_{tn}=i)\sim Categorical(\theta_{ik})$. Emission probabilities $\theta_{ik}$ and transition probabilities $\pi_{ijn}$ are assumed to be independent.

A hierarchical Bayesian approach was used for parameter estimation. By using the hierarchical Bayesian estimation, one general 'population' hidden Markov model is estimated via population-parameters, while heterogeneity between subjects is accommodated via individual-specific parameters. In addition to estimates of the “population” hidden Markov model, we are able to obtain individual-level estimates for both transition and emission probability matrices. Both latent states and observed ordinal outcomes are assumed to follow categorical distributions. Transition and emission probabilities are modeled through multinomial logit distribution. Hyper-prior distributions are used to account for population-level parameters that informed individual-level parameters. Estimation specifics are further detailed at <https://cran.r-project.org/web/packages/mHMMbayes/vignettes/estimation-mhmm.pdf> for interested readers.

We estimated 8 multilevel hidden Markov models with the number of states ranging from 2 to 10 respectively. We then fitted multilevel hidden Markov model to our data of 10 depressive symptoms from multiple subjects across 90 measurement occasions using “mHMMbayes” package in R. “mHMMbayes” uses a hybrid Metropolis within Gibbs sampler for model estimation. For each estimated model, we extracted the related AIC value. We found that AIC from all models with the number of states from 2 to 10 were 490.34, 452.50, 503.94, 557.64, 638.86, 704.82, 781.44, 869.96, 948.167 (as noted on footnote 1 in the main text and plotted in the figure here). Lower AIC represents better fit. Based on the model fit, we selected the final model with 3 hidden states. Results from the selected model are reported in the main text.


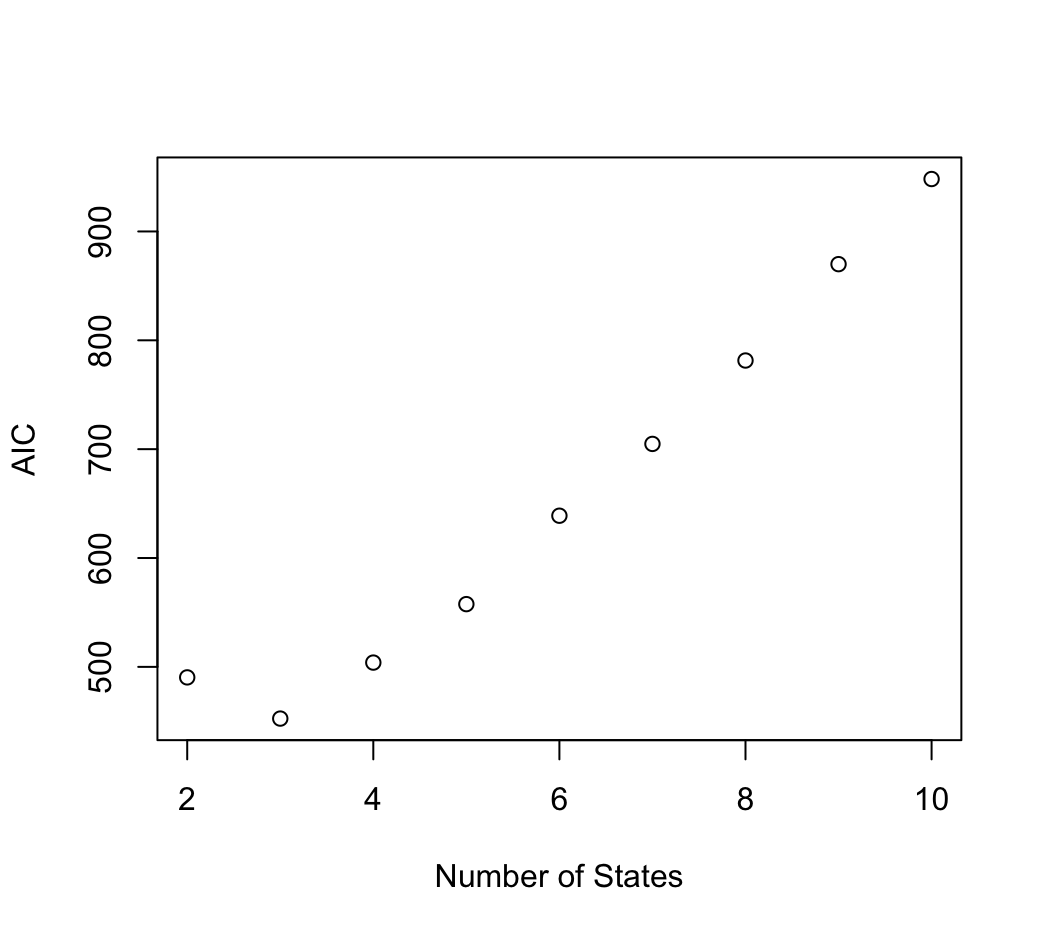

Supplement: 1 [file NIHMS1895870-supplement-1.docx]
